# Design, antimicrobial activity and mechanism of action of Arg-rich ultra-short cationic lipopeptides

**DOI:** 10.1371/journal.pone.0212447

**Published:** 2019-02-21

**Authors:** Federica Armas, Sabrina Pacor, Elena Ferrari, Filomena Guida, Thelma A. Pertinhez, Antonello A. Romani, Marco Scocchi, Monica Benincasa

**Affiliations:** 1 Department of Life Sciences, University of Trieste, Trieste, Italy; 2 Area Science Park, Padriciano, Trieste, Italy; 3 Department of Medicine and Surgery, University of Parma, Parma, Italy; 4 Department of Engineering and Architecture, University of Trieste, Trieste, Italy; 5 Transfusion Medicine Unit, AUSL-IRCCS of Reggio Emilia, Reggio Emilia, Italy; 6 Arta Peptidion srls, Parma, Italy; Nanyang Technological University, SINGAPORE

## Abstract

The increasing emergence of multidrug-resistant microorganisms represents one of the greatest challenges in the clinical management of infectious diseases, and requires the development of novel antimicrobial agents. To this aim, we *de novo* designed a library of Arg-rich ultra-short cationic antimicrobial lipopeptides (USCLs), based on the Arg-X-Trp-Arg-NH_2_ peptide moiety conjugated with a fatty acid, and investigated their antibacterial potential. USCLs exhibited an excellent antimicrobial activity against clinically pathogenic microorganisms, in particular Gram-positive bacteria, including multidrug resistant strains, with MIC values ranging between 1.56 and 6.25 μg/mL. The capability of the two most active molecules, Lau-RIWR-NH_2_ and Lau-RRIWRR-NH_2,_ to interact with the bacterial membranes has been predicted by molecular dynamics and verified on liposomes by surface plasmon resonance. Both compounds inhibited the growth of *S*. *aureus* even at sub MIC concentrations and induced cell membranes permeabilization by producing visible cell surface alterations leading to a significant decrease in bacterial viability. Interestingly, no cytotoxic effects were evidenced for these lipopeptides up to 50–100 μg/mL in hemolysis assay, in human epidermal model and HaCaT cells, thus highlighting a good cell selectivity. These results, together with the simple composition of USCLs, make them promising lead compounds as new antimicrobials.

## Introduction

The increasing emergence of multidrug-resistant pathogens has become one of the most pressing concerns in modern medicine. However, despite resources and energies invested to increase the knowledge of the resistance mechanisms, and in the search for ever more active drugs, the diffusion of resistance to antibiotics is currently proceeding faster than the discovery of new active compounds. Consequently, this problem has become an actual public health priority on a global scale, *de facto* reducing the possibility of an efficacious treatment [1, 2]. In particular, the incidence of methicillin-resistant *S*. *aureus* (MRSA), which often represents a serious problem for the management of skin and soft tissue infections [3, 4], is among the highest in Europe [5], while, in the last years, the frequency of *Enterococcus* vancomycin-resistance (VREF) has progressively increased all over the world.

Antimicrobial peptides (AMPs) constitute an effective component of natural immunity for host defense against microbial agents and are considered promising alternatives to conventional antibiotics. An alternative is represented by native lipopeptides, a class of antimicrobial molecules non-ribosomally produced by bacteria and fungi during the growth on various carbon sources [6, 7]. They consist of aliphatic acid attached to the N-terminus of a short cationic or anionic peptidic moiety of six to seven amino acids. Similarly to most AMPs, these lipopeptides act principally via perturbation of bacterial cell membranes [8], whilst their antifungal activity is mediated by their binding to the negatively charged membrane phosphatidylinositol and/or terminal sialic acid moieties [9, 10]. Despite much attention has been focused on naturally occurring lipopeptides, most of them are non-cell-selective molecules and cause severe cytotoxicity to mammalian cells [11, 12]. In parallel, a well-documented strategy aimed to increase AMPs efficacy is the conjugation of aliphatic fatty acids to N-terminus of positively charged peptides, generating new artificial lipopeptides with potent antifungal and antibacterial activities [13] and able to prevent antibiotic resistances, as reported for Lau-CKK-NH_2_ dimer and daptomycin [14]. Coherently, aliphatic acid conjugation to inactive or slightly active cationic peptides endowed them with antimicrobial activity [13]. In addition, many studies demonstrated that fatty acid attachment allows reducing the length of the peptidic chain by a compensative effect of its hydrophobic bulk [15, 16]. To sum up, synthetic lipopeptides have attracted considerable interest as an alternative to classical AMPs and native lipopeptides, due to their non-cytotoxic activity and high efficacy [17–19], together with an increased resistance towards proteolysis [15] and towards degradation in human plasma [20].

The abovementioned studies have revealed that a threshold of hydrophobicity and a defined structure of the peptide moiety are required for antimicrobial activity [13, 15, 16, 18]. Based on these premises, here we report the rational design and characterization of a new family of Arg-rich Ultra-Short Cationic Lipopeptides (USCLs), composed of only 4–6 amino acids conjugated to aliphatic acids with different chain lengths. Among a panel of predicted USCLs we determined the most favorable length of the aliphatic acid and optimized the peptide sequence for antimicrobial activity; further, the most effective ones were investigated for their cytotoxicity and mode of action. In our opinion, these results might be useful for the development of new antimicrobial drugs.

## Materials and methods

### Arg-rich ultra-short cationic lipopeptides (USCLs)

Arg-rich USCLs were *de novo* designed and screened by using *ad hoc* screening software whose core unit (Classification Unit) is represented by Feedforward Neural Networks, as extensively described in [21]. This screening software is property of Arta Peptidion, but any screening analyses of peptide sequences will be provided, upon request, by Arta Peptidion (info@artapeptidion.it). Briefly, the training dataset of AMPs was built over experimentally validated AMPs, available in the DBSAA database [22], APD3 [23], CAMP [24], whereas the UNIPROT database was used to construct the training non-AMPs dataset. The former dataset was then manipulated as follows: sequences longer than 30 amino acids in length, and those sharing 40% sequence identity, by using CD-HIT web server program [25], were filtered out. The latter dataset was built as follows: peptide sequences were downloaded from UniProt database [26], removing any entry that matches the following keywords: antimicrobial, antibiotic, antiviral, antifungal, toxin, effector or excreted. Similarly, sequences longer than 30 amino acid in length as well as those sharing 40% sequence identity, were filtered out. The input layer of neural network contains one node for each descriptor (physicochemical or structural parameter) used to define the environment of the sequence. To highlight the most representative properties of AMPs, 538 parameters were taken from AAIndex database [27]. By using different algorithm for features selection, 21 physicochemical and structural parameters were finally selected such as isoelectric point, amphipathicity, lipophilicity, charge, Boman index, or flexibility.

Finally, random sequences were generated using the Mersenne Twister algorithm, a 32-bit pseudo-random number generator developed by Makoto and Takuji [28], and then submitted to neural network for screening.

GenScript (Piscataway, NJ, USA) carried out the custom synthesis of USCLs by using high efficient solid phase peptide synthesis. The fatty acid was conjugated to the N-terminus of the peptide using the same procedure followed to attach protected amino acids to the growing peptide chain for synthesis. The purity (>90%), sequence and molecular weight of USCLs were assessed and provided by GenScript using RP-HPLC and ESI-MS. USCLs were dissolved in sterile water or DMSO at 10 mg/mL, according to manufacturer's indications. Abbreviation, molecular weight, and retention time in RP-HPLC of USCLs are summarized in **Table 1**. Their experimentally measured molecular weights were comparable with the corresponding theoretical values, and RP-HPLC retention times reliably reflected the hydrophobicity in aqueous solution.

**Table 1:** List of novel USCLs used in this study.

Sequence	Abbreviation	Molecular weight	Retention time^a^ (min)	Ref.
Hex-R**F**WR-NH_2_	Lp-F^Hex^	762.0	18.675	
Oct-R**F**WR-NH_2_	Lp-F^Oct^	788.6	17.974	
Dec-R**F**WR-NH_2_	Lp-F^Dec^	817.0	22.639	
Lau-R**F**WR-NH_2_	Lp-F^Lau^	845.3	23.592	
Myr-R**F**WR-NH_2_	Lp-F^Myr^	873.0	26.654	
Lau-R**A**WR-NH_2_	Lp-A	768.60	18.931	
Lau-R**C**WR-NH_2_	Lp-C	801.00	22.492	
Lau-R**G**WR-NH_2_	Lp-G	755.10	18.415	
Lau-R**H**WR-NH_2_	Lp-H	835.30	21.476	
Lau-R**I**WR-NH_2_	Lp-I	810.85	20.108	
Lau-R**L**WR-NH_2_	Lp-L	811.20	23.079	
Lau-R**M**WR-NH_2_	Lp-M	829.50	12.393	
Lau-R**R**WR-NH_2_	Lp-R	854.12	21.651	
Lau-R**S**WR-NH_2_	Lp-S	785.40	16.117	
Lau-R**V**WR-NH_2_	Lp-V	797.10	19.317	
Lau-**R**R**F**WR**R**-NH_2_	Lp-F^RR^	1157.60	22.087	
Lau-**R**R**I**WR**R**-NH_2_	Lp-I^RR^	1123.46	18.060	
^b^Lau-RRFW-NH_2_	Lau-RRFW	845.11	22.609	[36]
^b^Lau-RRIW-NH_2_	Lau-RRIW	811.09	20.957	[36]

Lau, lauric acid (C12); Myr, myristic acid (C14); Dec, decanoic acid (C10); Oct, octanoic acid (C8); Hex, hexanoic acid (C6)

^a^RP-HPLC retention time reliably reflects the hydrophobicity in aqueous solution.

^b^These molecules were obtained by shuffling of amino acids of Lp-F^Lau^ and Lp-I.

### Dynamic light scattering

Dynamic Light Scattering (DLS) technique was applied to evaluate USCLs aggregation in solution. DLS technique measures the intensity of the scattered light arising from particles in random Brownian motion in solution, detected at a fixed angle (90°) as a function of time. In a DLS measurement, scattering intensity fluctuations are correlated across small time spans, yielding a correlogram. By means of the correlation function, the analysis of the fluctuations of the scattered light intensity yields information about the hydrodynamic size of particles in suspension.

DLS measurements were carried out using Malvern Zetasizer μV instrument and, for calculating the particle size distribution, the non-negative least squares (NNLS) analysis was used. The intensity-weighted distribution analysis was used to extract the mean aggregate size from suitable data. Each measurement derived from three data acquisitions. Lp-I and Lp-I^RR^ were diluted in filtered PBS (0.2 μm Whatman syringe filter) at concentrations ranging from 1.5 μg/mL to 121.6 μg/mL (Lp-I) and 168.5 μg/mL (Lp-I^RR^).

### Surface plasmon resonance

Interaction studies between USCLs and model membranes were carried out using a X100 instrument (Biacore, GE Lifesciences) after immobilization of integral liposomes on a L1 sensor chip surface. Large unilamellar vesicles (LUVs) were prepared using dipalmitoylphosphatidylcholine (DPPC) and 1,2-dipalmitoylphosphatidylglycerol (DPPG) (4:1 vol/vol). Dry lipids were dissolved in 2:1 vol/vol chloroform/methanol, the solvent was removed, and resulting cake vacuum-dried overnight. The dry lipid cake was hydrated in PBS at a final concentration of 5 mM. The resulting multilamellar vesicle suspensions were then disrupted with several freeze-thaw cycles prior to extrusion through polycarbonate filters with 100 nm pores using a Mini-Extruder kit (Avanti Polar Lipids, Inc.).

The DPPC/DPPG LUVs suspension was diluted in PBS to 1 mM, and then injected three times onto the chip surface for 10 min, at flow rate of 5 μL/min. About 5000 Relative Units (RU) maximum was reached for all binding experiments.

To determine the binding of Lp-I and Lp-I^RR^ to LUVs, increasing concentrations of two USCLs (from 1.5 to 12 μg/mL) were sequentially injected, at a constant flow rate of 10 μL/min for a contact time of 540 sec, followed by a dissociation time of 1200 sec with PBS. Sensorgrams were obtained using BIAevaluation software v 1.1, and then elaborated using GraphPad v 6.04. Each experiment was repeated two times.

### Bacterial and fungal strains and growth conditions

Bacteria used in this study were *Escherichia coli* ATCC 25922 and O18K1H7, *Staphylococcus aureus* ATCC 25923 and ATCC 29213, *Pseudomonas aeruginosa* ATCC 27853, *Staphylococcus epidermidis* ATCC 12228, *Enterococcus faecalis* ATCC 29212, *Bacillus subtilis* DSM 4181, *Listeria monocytogenes* DSM 20600, *Salmonella typhimurium* ATCC 14028, *Acinetobacter baumannii* ATCC 19606 and 17978, *Klebsiella pneumoniae* ATCC 13883 and 700603, *Stenotrophomonas maltophilia* ATCC 13637 and *Burkholderia cenocepacia* J2315. In addition to reference strains, a clinical isolate of *E*. *faecalis* vancomycin-resistant (VREF, strain M), and three clinical isolates of *S*. *aureus* methicillin-resistant (MRSA, strains E, G and 11), kindly provided by Dr. Lucilla Dolzani [29], were used. All MRSA strains are resistant to GEN, IPM, OXA, PEN, CIP and CEF; the *S*. *aureus* E strain is also resistant to CLI and ERY. VREF strain is resistant to AMK, ATM, GEN, NET, PEN, TEC, TET, TOB and VAN.

For the assays, overnight bacterial cultures were diluted 1:30 in fresh Mueller-Hinton Broth (MHB, Difco) and incubated at 37°C with shaking for approximately 2h.

Fungal strains used were *Candida albicans* ATCC 90029 and SC5314. Fungi were grown on Sabouraud dextrose (SAB, Difco) agar plates at 30°C for 48h. The initial fungal inoculum was prepared by picking and suspending five colonies in 5 mL of sterile PBS.

### Evaluation of the antimicrobial activity

The antimicrobial activity of USCLs was determined by the Minimum Inhibitory Concentration (MIC) assay, according to the guidelines of CLSI (Clinical & Laboratory Standards Institute) and described in [30, 31]. Briefly, the assays were performed in 96-well microplates in appropriate broth (MHB or SAB). Some MIC assays were performed in Cation-Adjusted Mueller-Hinton Broth (CA-MHB), containing 20–25 mg/L of calcium and 10–12.5 mg/L of magnesium. For bacteria, a mid-log phase bacterial suspension at 2.5 × 10^5^ CFU/mL in MHB was used, and the microplates incubated at 37°C for 24h. The fungal suspension in SAB broth was used at a final concentration of 5 × 10^4^ cells/mL. Microplates were then incubated at 30°C for 48h. The MIC value was taken as the lowest concentration of USCLs resulting in the complete inhibition of visible bacterial or fungal growth after appropriate incubation time. Results derive from at least three independent experiments carried out in duplicate.

Bacterial growth inhibition tests were performed using mid-log phase bacterial cultures diluted in MHB to 1 × 10^6^ CFU/mL, in presence of different concentrations of USCLs. Bacterial growth was followed at 37°C with intermittent shaking for 4h by measuring every 10 min the absorbance at 620 nm with a microplate reader (Tecan Trading AG, Switzerland).

### Flow cytometric analysis

The flow cytometric assays were performed with Cytomics FC 500 instrument (Beckman-Coulter, Inc., Fullerton, CA). Integrity of bacterial cell membrane was assessed by measuring the propidium iodide (PI) uptake by flow cytometry, as described in [32]. Briefly, mid-log phase bacterial cultures, diluted at 1 × 10^6^ CFU/mL in MHB, were incubated at 37°C for different times with increasing concentrations of Lp-I and Lp-I^RR^. PI was added to all samples at a final concentration of 10 μg/mL. At the end of the incubation, the bacterial cells were analysed by flow cytometry. The permeabilization kinetics was followed up to 60 min because prolonged time of treatment could allow detecting membrane lysis as indirect effects of cellular death.

Data analysis was performed with the FCS Express3 software (De Novo Software, Los Angeles, CA). Data are expressed as mean ± SEM.

### Scanning electron microscopy (SEM)

Mid log phase *S*. *aureus* ATCC 25923 were diluted at 10^7^ CFU/mL in MH broth, and incubated at 37°C with 30 μg/mL Lp-I and Lp-I^RR^. After 60 min the cells were pelleted by centrifugation at 3000g for 5 min, followed by washing twice with PBS. The cells were then fixed at room temperature for 2h in 2.5% glutaraldehyde in PBS. After fixation for 2h at room temperature, bacteria were collected on a nitrocellulose filter with pore size of 0.2 μm, rinsed twice with PBS for 15 min, and subsequently dehydrated in ethanol solutions of increasing concentrations (30%, 50%, 70%, 80% and 100%) for 10 min at each concentration. After complete dehydration, all samples were covered with an appropriate volume of hexamethyldisilazane (Sigma) as a drying agent. Subsequently, the samples were sputtered with gold (Sputter Coater K550X, Emitech, Quorum Technologies Ltd, UK) and immediately analyzed with Scanning Electron Microscope (Quanta250 SEM, FEI, Oregon, USA) operated in secondary electron detection mode. The working distance and the accelerating voltage were adjusted in order to obtain a suitable magnification.

### In vitro toxicity assays

The hemolysis of human red blood cells (hRBCs) was performed by using a protocol previously described in [18]. Erythrocytes were isolated from buffy coats of informed healthy donors (in accordance with the ethical guidelines and approved from the ethical committee of the University of Trieste). Briefly, hRBCs were isolated via centrifugation, washed three times with PBS, and then suspended to 8% (v/v) in PBS. 100 μL hRBC suspensions were added into each well of a 96-well microplate. To each well was added 100 μL lipopeptide solution at different concentrations (the final concentration of hRBC suspension was 4% v/v), and the plates were incubated for 1 h at 37°C and centrifuged at 1000 g for 10 min. 100 μL aliquots of supernatant were transferred to fresh 96-well microplate, and the release of hemoglobin was monitored by measuring the absorbance at 540 nm with Tecan microplate reader (Tecan Trading AG, Switzerland). 0% and 100% hemolysis was determined in PBS and 1% Triton X-100, respectively.

Cytotoxicity was also determined by the MTT assay using human epidermal keratinocytes HaCaT cells (DKFZ, Eppelheim, Germany). HaCaT cells were cultured in Dulbecco’s Modified Eagle’s Medium (DMEM) High-glucose (Euroclone) supplemented with 10% fetal bovine serum (FBS), 2 mM glutamine, 100 U/mL penicillin and 100 μg/mL streptomycin at 37°C in a humidified 95% air and 5% CO_2_ atmosphere. Cell passage of confluent cells was performed once per week.

Cells were seeded in 96-wells plates at a density of 2 × 10^4^ cells/well and, the day after, exposed to different concentrations of Lp-I and Lp-I^RR^ USCLs for 1h or 24h in complete medium. After the 1h-incubation with USCLs, samples were washed with PBS and maintained for further 23h in fresh complete medium. Ten μL of MTT (5 mg/mL in PBS, Sigma-Aldrich) were then added to each well during the last 3h of incubation; the insoluble crystals were solubilized by 100 μL of 10% IGEPAL in 0.01N HCl (Sigma-Aldrich), and the plates were incubated overnight at 37°C. The absorbance was measured by a Chameleon plate reader (Hidex) at 544 nm. Data are reported as % of control and are the mean ± SEM of 3 independent experiments performed in triplicate.

### Toxicity study with tissue-based EpiDerm

EpiDerm tissues, consisting of normal human-derived epidermal keratinocytes cultured to form a multilayered highly differentiated model of the human epidermis, were obtained from MatTek Corporation. The manipulation of tissues and the experimental procedure for the toxicity test with Lp-I and Lp-I^RR^ USCLs were carried out according to the supplier’s protocol.

Prior to use, tissues were removed from the agarose shipping tray, placed into a six-well plate containing 0.9 mL of the Dulbecco’s Modified Eagle’s Medium (DMEM) provided by MatTek Corporation, and incubated for 1h at 37°C and 5% CO_2_.

After this initial incubation, EpiDerm inserts were transferred to the wells of a 6-well plate containing 0.9 mL DMEM and pre-incubated in a 5% CO_2_ incubator overnight.

After pre-incubation, tissues were transferred in a 6-well plate containing 0.9 mL of pre-warmed medium for each well; 100 μL of Lp-I and Lp-I^RR^ samples (stock 100 μg/mL) were applied directly on to the surface of EpiDerm tissues and maintained for 1h or 24h at 37°C and 5% CO_2_. A 5% SDS solution and PBS were used, respectively, as positive and negative control. Three tissues were used for each condition. For 1h-treated tissues, the tested compounds were removed by repeated rinsing with PBS, and tissues were transferred to the new plates with 0.9 mL of DMEM and maintained overnight in a 5% CO_2_ incubator prior to the viability assay.

For viability testing, after washes with PBS, all tissues were transferred to a 24-well plate containing 300 μL/well of MTT stock solution (1 mg/mL in DMEM). Samples were incubated in a 5% CO_2_ incubator for 3h and insoluble formazan products of MTT were extracted with 2 mL isopropanol that was added to each well. A 200 μL aliquot of the isopropanol extract was transferred to a well of a 96-well plate and the optical density (OD) was measured at 544 nm with a Chameleon plate reader (Hidex), using 200 μL of isopropanol as blank.

### Statistical analysis

Significance of differences among groups was assessed by using the program InStat (GraphPad Software Inc.) and performed by an analysis of variance between groups (ANOVA) followed by the Student Newman-Keuls post-test. Values of p < 0.05 were considered statistically significant.

## Results

### Design and characterization of the antimicrobial activity of ultra-short cationic lipopeptides (USCLs)

An *ad hoc* screening software was used to mine putative antimicrobial peptides (AMPs). The core is represented by the screening unit that uses the response of five neural networks to pick out potential AMP sequences, as extensively described in [21]. To identify the optimal sequence for obtaining active USCLs, we initially screened a randomly generated tetrapeptides considering their net charge, hydrophobicity, occurrence at least of one bulky aromatic residue, as reported in Materials and Methods. The sequence Arg-Phe-Trp-Arg (RFWR) satisfied all parameters and, additionally, was classified as potential AMP by all five neural networks.

In order to assemble active lipopeptide molecules, this peptide was amidated at the C-terminus and coupled to lipophilic alkyl chains with increasing number of carbon atoms (from C6 to C14) at N-terminus (**Table 1**). The antimicrobial activity of this group of USCLs was tested against three reference bacterial strains, and the results are reported in **Table 2**.

**Table 2:** Minimum inhibitory concentration (MIC^a^, in μg/mL) of USCLs against reference bacterial strains.

Compound	*S*. *aureus* ATCC 25923	*E*. *coli* ATCC 25922	*P*. *aeruginosa* ATCC 27853
Lp-F^Hex^	>400	>400	>400
Lp-F^Oct^	100	100–50	100
Lp-F^Dec^	25–12.5	25	50
Lp-F^Lau^	6.25–3.125	12.5–6.25	25–12.5
Lp-F^Myr^	6.25	50–25	50
Lp-A	6.25	50–25	25
Lp-C	50	200	200
Lp-G	6.25	25	50
Lp-H	6.25	25	100
Lp-I	3.125–1.56 (**3.125**)	6.25 (**6.25**)	12.5 (**25**)
Lp-L	6.25–3.125	25	25
Lp-M	6.25–3.125 (**6.25**)	6.25 (**12.5**)	12.5 (**25**)
Lp-R	3.125	12.5	25
Lp-S	6.25	25	25
Lp-V	6.25 (**6.25**)	12.5 (**12.5**)	12.5 (**25**)
Lp-F^RR^	3.125	12.5	12.5
Lp-I^RR^	3.125 (**3.125**)	12.5 (**12.5**)	12.5 (**25**)
Lau-RRIW^b^	3.125	12.5	12.5
Lau-RRFW^b^	3.125	12.5–6.25	12.5–6.25

^a^MIC was defined as the lowest concentration of compound that prevented bacterial visible growth after incubation for 24h at 37°C. Results derive from at least three independent experiments carried out in duplicate. MIC values are given as single value when replicates gave identical results, and as two values when replicates differed by one well. MIC values in boldface were obtained in Cation-Adjusted Mueller-Hinton Broth.

^b^These molecules were obtained by shuffling of amino acids of Lp-I and Lp-F^Lau^.

MIC values obtained with Lp-F^Hex^, Lp-F^Oct^, Lp-F^Dec^ and Lp-F^Lau^ evidenced a clear correlation between the length of the acyl chain and the antibacterial activity. In particular, the conjugation with lauric acid (C12, indicated as “Lau”) strongly enhanced the antimicrobial activity against all the tested bacteria in comparison with the molecules presenting a shorter acyl chain (C6, C8 and C10). The MIC values of Lp-F^Dec^, Lp-F^Oct^ and Lp-F^Hex^ increased from 4 to 128 fold against *S*. *aureus*, and from 4 to 32 against *E*. *coli* and *P*. *aeruginosa* compared to that of Lp-F^Lau^. MIC values obtained with Lp-F^Myr^ indicated that a further increase of acyl chain length slightly reduces the efficacy of the molecule, suggesting that the C12 length is optimal to confer a relevant antibacterial activity.

Then, by considering the well-known importance of cationic residues and bulky aromatic ones for AMP's activity, the relevance of Phe in Lp-F^Lau^ was investigated by evaluating the antimicrobial activity of a series of USCLs obtained by the substitution of this residue with other amino acids (**Table 2**). Overall, these molecules showed a remarkable activity against *S*. *aureus*, with MIC values ranging from 1.56 to 6.25 μg/mL. The most active compounds against this microorganism, Lp-I, Lp-L, Lp-M and Lp-R, showed a good efficacy also against *E*. *coli* and *P*. *aeruginosa* strains, with a MIC range of 6.25–25 μg/mL against the former, and 12.5–25 μg/mL against the latter. Only Lp-C, classified by the screening as non-AMP, was scarcely active against the three reference strains.

It is worth to note that the Phe substitution with other hydrophobic residues such as Ile, Leu or Val, or with Arg basic residue did not drastically modify the antibacterial activity of Lp-F^Lau^ against all strains. On the contrary, the substitution Phe→Gly or Phe→His reduced the efficacy against *P*. *aeruginosa*.

Next, in order to evaluate if the increase of the length and charge improve the activity, the selected peptide sequences of Lp-F^Lau^ and Lp-I were lengthened by one Arg residue at N-terminus and one Arg at C-terminus, generating the lipo-hexapeptides Lp-F^RR^ and Lp-I^RR^. These two novel molecules did not show improved activity against the three reference strains (**Table 2**), indicating that an increase of the positive charge and length of the peptide moiety did not lead to more potent compounds.

Interestingly, the activity of Lp-I, Lp-M, Lp-V and Lp-I^RR^, selected among the most effective USCLs against the reference strains, was maintained even in Cation-Adjusted Mueller-Hinton Broth, characterized by higher concentrations of divalent cations that generally contribute to membrane stabilization (**Table 2**, values in boldface) [33, 34].

The most active molecules (Lp-I, Lp-M, Lp-R, Lp-F^RR^ and Lp-I^RR^, see **Table 2**) were further assayed against a panel of Gram-positive (**Table 3**), Gram-negative bacteria and fungi (**Table 4**). The antimicrobial activity resulted more pronounced against Gram-positive bacteria, with MIC values ranging from 1.56 to 6.25 μg/mL for all tested strains, except for *E*. *faecalis* that showed a slightly lower susceptibility (**Table 3**). In addition, the good activity of these selected USCLs against clinical MRSA isolates (that were also resistant to different classes of antibiotics) and against a VREF strain, suggests that their mechanism of action is likely different from that of methicillin and vancomycin (**Table 3**).

**Table 3:** Minimum inhibitory concentration (MIC^a^, in μg/mL) of selected USCLs against a panel of Gram-positive bacterial strains.

Compound	*S*. *aureus* ATCC 29213	*S*. *aureus* G^b^	*S*. *aureus* E^b^	*S*. *aureus* 11^b^	*S*. *epidermidis* ATCC 12228	*E*. *faecalis* ATCC 29212	*E*. *faecalis* M^c^	*L*. *monocytogenes* DSM 20600	*B*. *subtilis* DSM 4181
Lp-I	3.125	3.125	3.125	3.125	6.25–3.125	6.25	6.25	1.56	3.125
Lp-M	6.25–3.125	6.25	6.25	6.25	3.125	6.25	12.5	3.125	3.125
Lp-R	3.125–1.56	3.125	3.125	3.125	3.125–1.56	6.25	12.5	3.125–1.56	1.56
Lp-F^RR^	3.125–1.56	3.125	3.125	3.125	3.125–1.56	6.25	6.25–12.5	1.56	1.56
Lp-I^RR^	3.125–1.56	3.125	3.125	6.25	3.125	12.5	12.5	3.125	1.56
Lau-RRIW^d^	3.125	3.125	3.125	6.25–3.125	6.25–3.125	6.25	6.25	3.125	3.125
Lau-RRFW^d^	3.125	6.25	3.125	6.25	6.25–3.125	6.25	12.5–6.25	3.125	6.25–3.125

^a^MIC was defined as the lowest concentration of compound that prevented visible growth after incubation for 24h at 37°C. Results derive from at least three independent experiments carried out in duplicate. MIC values are given as single value when replicates gave identical results, and as two values when replicates differed by one well.

^b^*S*. *aureus* isolates were MRSA strains.

^c^*E*. *faecalis* was VREF strain.

^d^These molecules were obtained by shuffling of amino acids of Lp-I and Lp-F^Lau^.

**Table 4:** Minimum inhibitory concentration (MIC^a^, in μg/mL) of selected USCLs against a panel of Gram-negative bacteria and *C*. *albicans* strains.

Compound	*E*. *coli* O18K1H7	*S*. *typhimurium* ATCC 14028	*K*. *pneumoniae* ATCC 13883	*K*. *pneumoniae* ATCC 700603	*A*. *baumannii* ATCC 19606	*A*. *baumannii* ATCC 17978	*S*. *maltophilia* ATCC 13637^c^	*B*. *cenocepacia* J2315^c^	*C*. *albicans* ATCC 90029	*C*. *albicans* SC 5314
Lp-I	12.5	25	100	50	25	25	6.25	12.5	3.125	12.5
Lp-M	25	100–50	>100	100	25	25	12.5	25	3.125	12.5
Lp-R	25	100	>100	100	25–12.5	25–12.5	12.5	50	1.56	50
Lp-F^RR^	25	25	25	25	25–12.5	25	12.5	12.5	1.56	100
Lp-I^RR^	25	50–25	100	50	25	25	12.5	12.5	1.56	200
Lau-RRIW^b^	12.5	25	100–50	50–25	12.5	25	12.5	6.25	3.125	12.5
Lau-RRFW^b^	12.5	12.5–6.25	50	25	12.5	12.5	6.25	12.5	3.125	12.5

^a^MIC was defined as the lowest concentration of compound that prevented visible growth after incubation for 24h at 37°C, or 48h at 30°C, respectively for bacteria and fungi. Results derive from at least three independent experiments carried out in duplicate. MIC values are given as single value when replicates gave identical results, and as two values when replicates differed by one well.

^b^These molecules were obtained by shuffling of amino acids of Lp-I and Lp-F^Lau^.

^c^For slow-growing *S*. *maltophilia* and *B*. *cenocepacia*, reported values correspond to MIC after 48h of incubation.

The compounds were also active against Gram-negative bacteria, although with MIC values ≥ 12.5 μg/mL (**Table 4**). A good result was obtained with Lp-I against *S*. *maltophilia* and *B*. *cenocepacia* reference strains, with MICs of 6.25 and 12.5 μg/mL, respectively. The efficacy displayed against *B*. *cenocepacia* is a very promising result, which will require further investigations, in particular by considering the serious problem about the natural antibiotic resistance of *Burkholderia* spp. [35].

Lp-I, Lp-M and Lp-R showed good activity against two different reference strains of *C*. *albicans*, with MIC values ranging from 1.56 to 50 μg/mL, while a dramatic reduction of susceptibility to the cationic Lp-R, Lp-F^RR^ and Lp-I^RR^ lipo-hexapeptides was observed with *C*. *albicans* SC5314, suggesting that an increase in length and/or in positive charge of these USCLs has a detrimental effect on the activity against this *Candida* strain.

The most effective lipopeptides Lp-I and Lp-I^RR^, with four and six amino acid residues respectively, were selected for growth kinetics and cytotoxicity studies, and to investigate their mechanism of action.

### Effects of USCLs on bacterial growth rate

**Fig 1:**
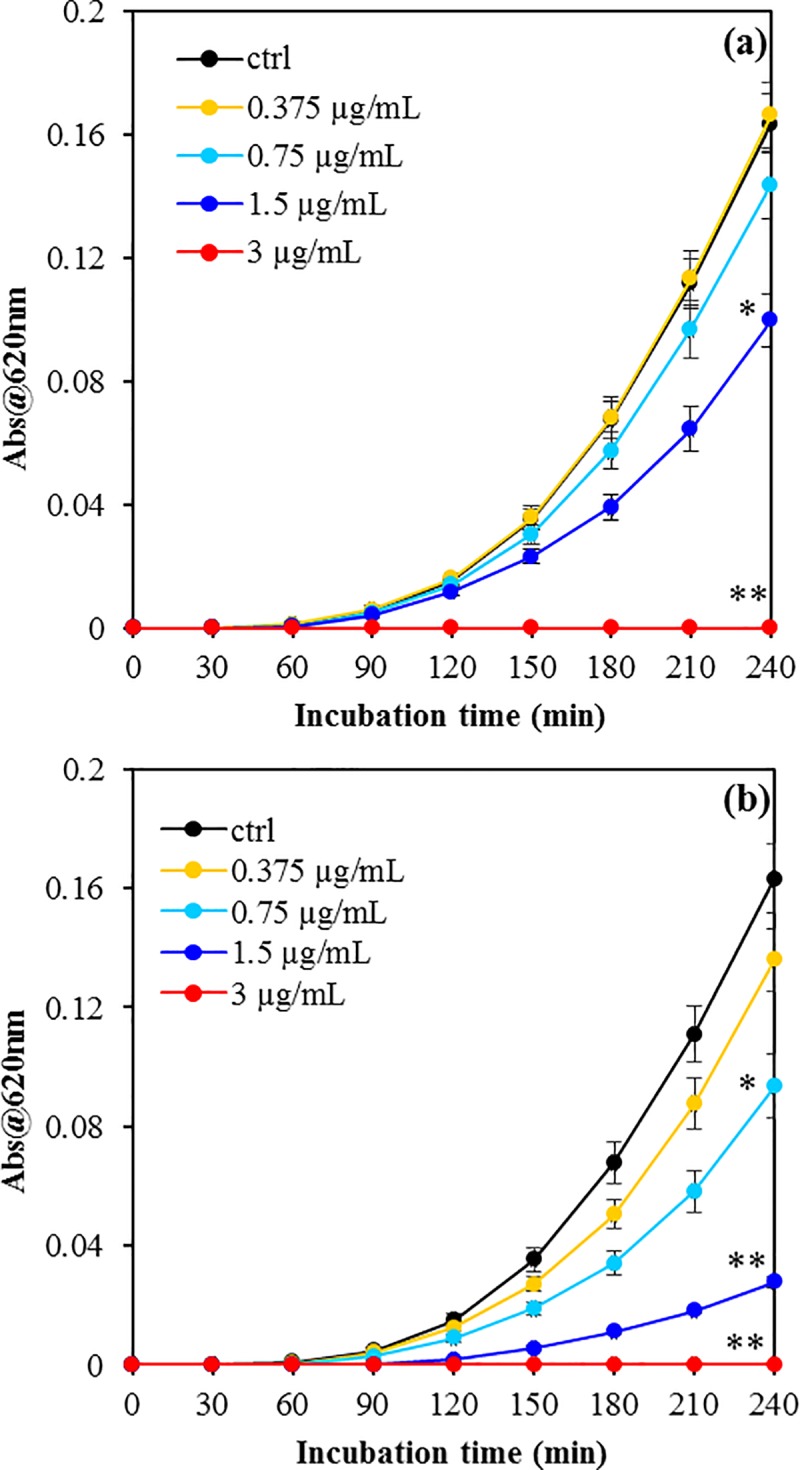
Growth kinetics of *S*. *aureus* ATCC 25923 in presence of different concentrations of USCLs. Bacterial suspensions (1×10^6^ cells/mL) was grown up to 4h in presence of indicated concentrations of Lp-I (**a**) and Lp-I^RR^ (**b**), and the OD_620_ was measured every 10 min. The results are the mean ± SEM of three independent experiments. *p < 0.05 *vs* untreated cells (ctrl) at 4h incubation time; **p < 0.0001 *vs* untreated cells (ctrl) at 4 h incubation time (Student-Newman-Keuls Multiple Comparisons Test, ANOVA).

We investigated the antibacterial activity of Lp-I and Lp-I^RR^ by a short-term growth inhibition assay, using sub MIC concentrations (**Fig 1**).

Lp-I used at ⅛ or ¼MIC value did not show any significant effect on *S*. *aureus* ATCC 25923 growth rate; nevertheless, it was sufficient to increase the Lp-I concentration to ½MIC (1.5 μg/mL) to obtain a partial inhibition of bacterial growth (**Fig 1**). For a complete inhibitory effect it was required a concentration corresponding to MIC value (3 μg/mL) (**Fig 1A**). Conversely, the growth rate in presence of increasing concentrations of Lp-I^RR^ showed a gradual and concentration-dependent inhibitory effect (**Fig 1B**). A significant inhibitory effect of bacterial growth with Lp-I^RR^ was already observed at ¼MIC and, by increasing the concentration up to MIC value, the effect became more pronounced. Overall, these results indicated that even at sub MIC concentration, these compounds are able to decrease the bacterial growth rate.

### Evaluation of the cytotoxicity

To evaluate the potential of Lp-I and Lp-I^RR^ to damage eukaryotic membrane, their capacity to lyse a 4% (v/v) suspension of hRBC was assayed by colorimetric hemolysis assay, and results were shown in Supporting Information (**S1 Fig**). Briefly, Lp-I and Lp-I^RR^ induced a significant lytic effect, respectively, at 50 and 100 μg/mL with a 50–60% hemolysis. At 25 μg/mL, both molecules did not display any significant membrane effect on hRBC (≤ 10%), and the percentage reduced to zero at even lower concentrations.

**Fig 2:**
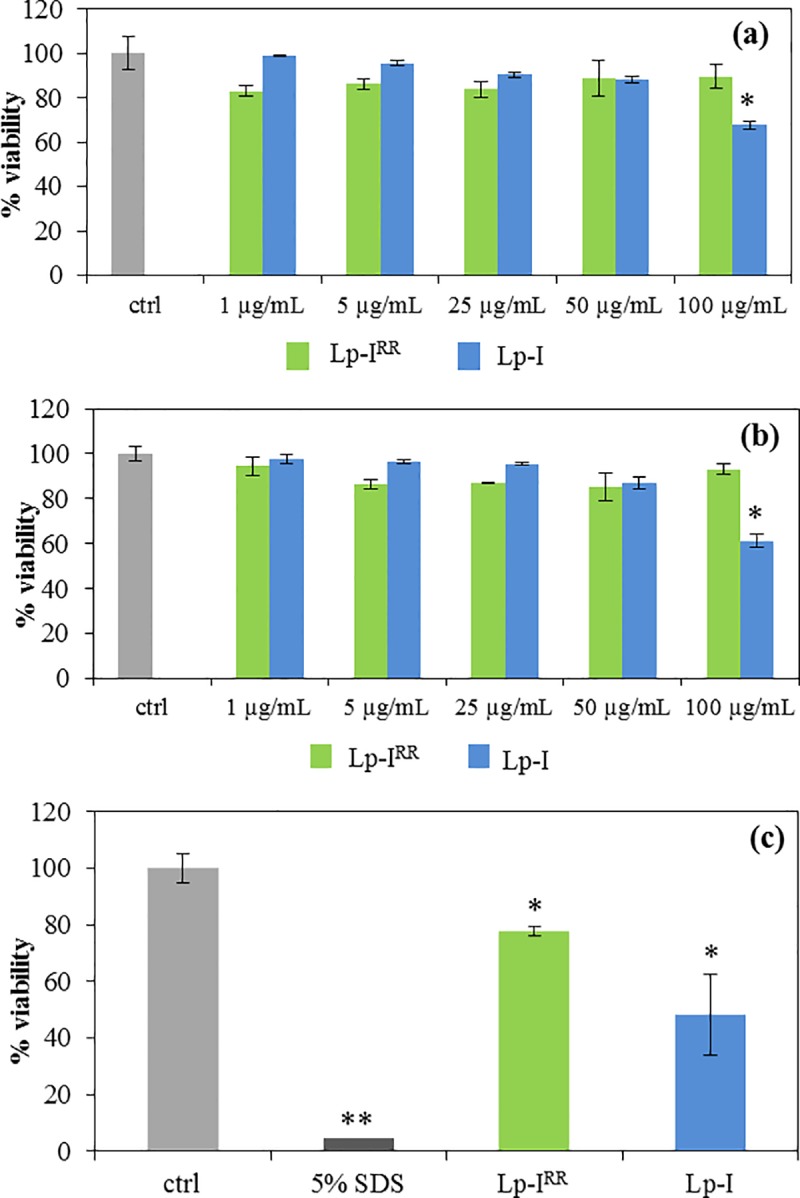
Effects of Lp-I and Lp-I^RR^ on human epidermal keratinocytes HaCaT cells and the human epidermal model EpiDerm. The cell viability is expressed as a percentage of the OD measured on untreated cells (ctrl) assumed as 100% viability. Cytotoxic activity on HaCaT cells was evaluated after 1h (**a**) or 24h (**b**) incubation with indicated concentrations of USCLs. (**c**) The cytotoxicity with EpiDerm test was evaluated after 1h incubation with 100 μg/mL of both USCLs; 5% SDS was used as positive control. Each value represents the mean ± SEM of 3 experiments performed in triplicate. *p < 0.05 *vs* untreated cells (ctrl); **p < 0.0001 *vs* untreated cells (ctrl) (Student-Newman-Keuls Multiple Comparisons Test, ANOVA).

We also evaluated the cytotoxicity of Lp-I and Lp-I^RR^ on human epidermal keratinocytes HaCaT cells also using MTT assay. The two compounds were incubated with the cells for 1h (**Fig 2A**) or 24h (**Fig 2B**) at concentrations ranging from 1 to 100 μg/mL. Lp-I^RR^ did not cause any significant reduction in cell viability even when used at 100 μg/mL, whilst Lp-I at the same concentration reduced cell viability to 30% and 40%, after 1h and 24h treatment, respectively. Lp-I did not show any cytotoxicity at lower concentrations. These results indicated that both lipopeptides affects the membrane integrity of hRBC and the viability of keratinocytes only at concentrations well above their MIC values.

The potential cytotoxic effect of Lp-I and Lp-I^RR^ was also evaluated by using the Epiderm test, a reconstructed human epidermidis tissue, constituted by a cell multilayer epidermal barrier, endowed with a stratum corneum, an intermediate spinous and granular layers, and a basal cell layer [37]. Lp-I^RR^ at 100 μg/mL reduced the cell viability by ~20%, whilst, at the same concentration, Lp-I decreased cell viability by 50% (**Fig 2C**). However, the cytotoxic concentrations of USCLs were well above the MIC values. The more hydrophobic character of Lp-I could favor its diffusion throughout the thickness of the epithelial barrier, causing higher cytotoxic effects than Lp-I^RR^.

### Analysis of the aggregation potential of Lp-I and Lp-I^RR^

According to the analysis of Nasompag *et* al. [38], we investigated the aggregation potential of Lp-I and Lp-I^RR^ in solution by means of Dynamic Light Scattering.

Starting with 10 μM sample concentration (corresponding to 8.1 μg/mL for Lp-I and 11.2 μg/mL for Lp-I^RR^) and up to 150 μM, the derived size distributions unveiled the presence of polydisperse populations of aggregates, with mean diameter varying in the range of 225–594 nm, with the higher values being attributed to Lp-I aggregates (**S2 Fig**). As an example, at 100 μM, Lp-I^RR^ size distribution highlighted a monomodal dispersion of aggregates with mean diameter of 321.2 nm ± 43.7, while the Lp-I one revealed a population of aggregates with mean diameter of 474.2 nm ± 69.4, coexisting with larger and probably sedimenting aggregates (**S2 Fig**). These sedimenting particles were also detected in samples at different concentrations, together with a more defined population of aggregates. The higher size of Lp-I aggregates extracted at concentrations ≥10 μM (**S2 Fig**) correlates with the minor positive charge and length of its peptide moiety. At the lowest tested concentrations of 1.5, 3 and 6 μg/mL, corresponding to the range of MICs, DLS measurements detected aggregation of both the lipopeptides. However, their signal strength and derived data quality resulted too poor for size distribution analysis, but still suggestive of polydisperse and heterogeneous aggregates in solution. In fact, the analysis of the DLS measured correlograms suggested the presence of polydisperse aggregates of a similar order of magnitude as those obtained at higher concentrations; nevertheless, their signal is considerably disturbed by the co-presence of heterogeneous and dynamic macroaggregates, making problematic the extraction of a size distribution.

### USCLs-membrane model interaction

**Fig 3:**
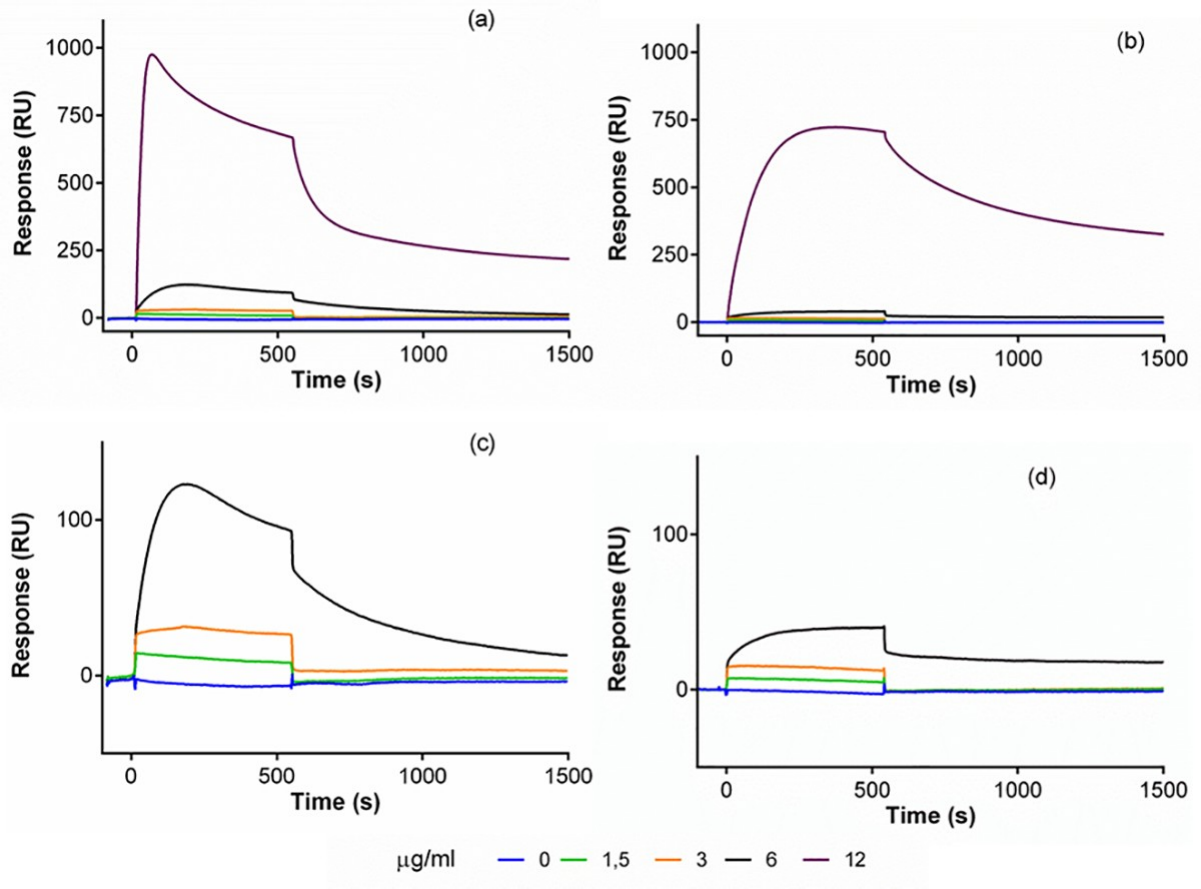
Binding sensorgrams for USCLs onto immobilized LUVs. Solutions with increasing concentrations (1.5, 3, 6 and 12 μg/mL) of Lp-I (**a, c**) and Lp-I^RR^ (**b, d**) were injected onto L1 sensor chip surface with immobilized LUVs (DPPC/DPPG 4:1 v/v). The baseline was adjusted to zero at each injection.

The interaction of Lp-I and Lp-I^RR^ with a membrane model constituted by immobilized large unilamellar vesicles (LUVs) with DPPC/DPPG (4:1 v/v), was investigated with surface plasmon resonance. The profile of binding curves in **Fig 3** indicated that both USCLs were able to bind this membrane model already at the lowest concentration (1.5, 3 and 6 μg/mL, **Fig 3C** and **3D**), showing a gradual increase of the response units (RU value) of the binding curves with rising concentrations of lipopeptides. In particular, at 12 μg/mL the binding curves displayed a dramatic increase in the signal not corresponding to the proportional increase of concentration (**Fig 3A** and **3B**), suggesting a possible self-aggregation or oligomerization [39] for both the molecules. This increase was more pronounced for Lp-I in agreement with the larger size of aggregates formed by this molecule than Lp-I^RR^ (see **S2 Fig**). Furthermore, it is worth to note that only for Lp-I, the shape of this sensorgram changes, with a loss of RU during the injection, suggesting a putative disaggregation of membrane-bound aggregates, without disruptive effect of the LUVs [40].

During the dissociation phase, the binding curve did not return to the baseline after wash with PBS, indicating a stable and irreversible binding complex for both USCLs with LUVs starting from 6 μg/mL. The membrane interaction capability of Lp-I and Lp-I^RR^ was also predicted by full-atomistic molecular dynamics which suggests, for both USCLs, a very rapid insertion of their acyl chains (within the first 20 ns) in the membrane model, and a faster insertion into the membrane of Lp-I peptide moiety as compared to Lp-I^RR^ (**S3 Fig**).

### Effects of USCLs on bacterial membrane

We investigated the effects of Lp-I and Lp-I^RR^ on bacterial membrane integrity by Propidium Iodide (PI) uptake assay.

**Fig 4:**
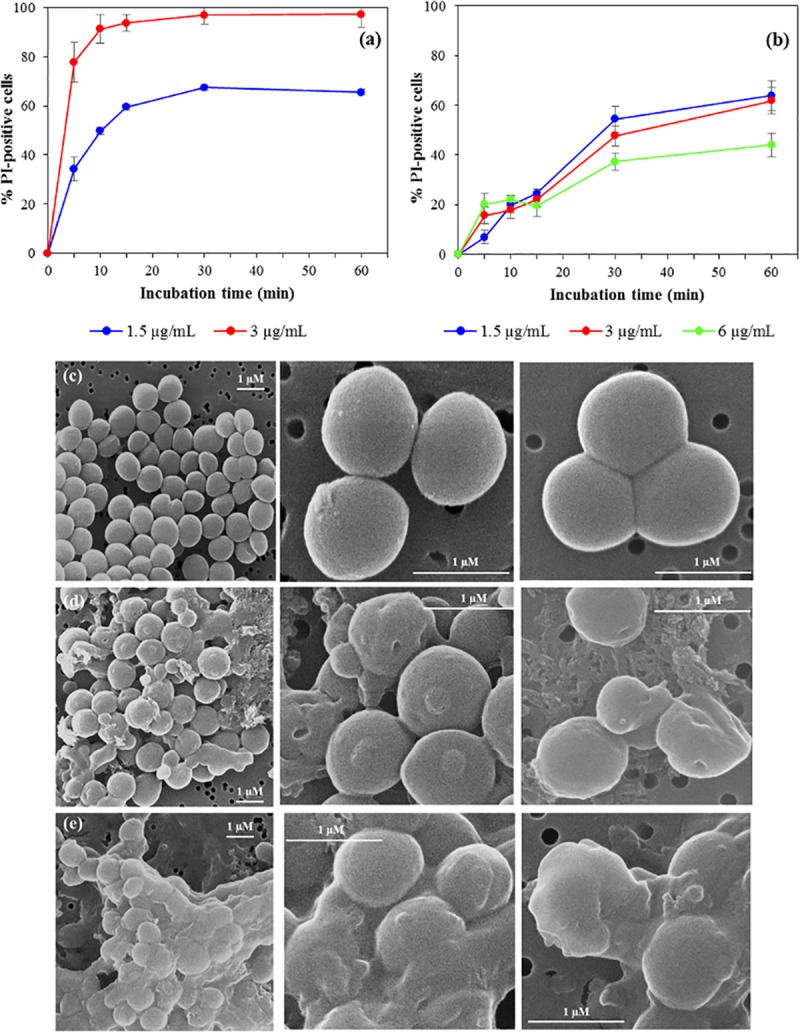
Evaluation of membrane-damaging activity of Lp-I and Lp-I^RR^ on *S*. *aureus* ATCC 25923 by PI-uptake assay and scanning electron microscopy. The permeabilization assay with Lp-I (**a**) and Lp-I^RR^ (**b**) on *S*. *aureus* cells has been performed in MHB. Bacterial cells (1×10^6^ CFU/mL) were incubated, for different incubation times, with USCLs at the concentration equal to their MIC, ½ MIC or 2×MIC. % PI-positive: percentage of propidium iodide positive cells. The background level of permeabilized cells, obtained with untreated samples, was always lower than 2% and was subtracted to the corresponding USCL-treated sample. Data are a mean ± SEM of four independent experiments. Scanning electron microscopy of 10^7^ CFU/mL *S*. *aureus* cells untreated (**c**) or after 60 min incubation at 37°C with 30 μg/mL of Lp-I (**d**) or Lp-I^RR^ (**e**).

Both USCLs permeabilized the *S*. *aureus* membrane at concentrations near their MICs (**Fig 4A** and **4B**). At MIC value (3 μg/mL), Lp-I caused permeabilization of >90% of bacterial cells after only 15-min incubation (**Fig 4A**), whilst the same concentration of Lp-I^RR^ resulted in ~60% of PI-positive cells after 60-min incubation (**Fig 4B**). By using Lp-I and Lp-I^RR^ at ½MIC, the results did not change, showing a rapid membrane damage for the former (~ 60% PI-positive cells after 15-min incubation), and a delayed effect for the latter, which reached the same percentage of damaged cells only after 60-min incubation. The increase of the Lp-I^RR^ concentration at 2×MIC did not increase the percentage of damaged cells (**Fig 4B**). The lytic effect of Lp-I and Lp-I^RR^ was also demonstrated against *E*. *coli* at concentrations near their MIC values (**S4 Fig**). In particular, both USCLs caused an appreciable percentage of damaged cells (~70%) after 30-min incubation with 12 μg/mL. Reducing the concentration at 6 μg/mL, Lp-I did not cause any effect, whilst Lp-I^RR^ induced ~40% of bacteria damaged after 60-min treatment (**S4 Fig**).

The effect of Lp-I and Lp-I^RR^ on *S*. *aureus* viability was also evaluated by using the same concentrations and incubation time causing the membrane permeabilization (**Fig 4A** and **4B**). The significant reduction of the number of viable cells after USCLs treatment (**S5 Fig**), indicates that membrane damage induced by Lp-I and Lp-I^RR^ was lethal for bacterial cells.

We also investigated the effect of the USCLs treatment on *S*. *aureus* morphology, by using scanning electron microscopy (SEM) (**Fig 4C**, **4D** and **4E**). The treatment with Lp-I and Lp-I^RR^ induced dramatic morphological changes on the bacterial surface: untreated cells had a normal, smooth surface (**Fig 4C**), whilst the surface of cells treated with Lp-I (**Fig 4D**) and Lp-I^RR^ (**Fig 4E**) became roughening and blebbing. Moreover, treated cells with Lp-I often showed holes in their surface, and cellular debris likely arising from cell lysis. Indeed, these morphological alterations of bacterial surface indicated a membrane-damaging activity of these USCLs, in agreement with data obtained in PI-uptake assays.

## Discussion

The problem of antibiotic-resistance might be faced and partially solved by the introduction of new antimicrobials in clinics. Although AMPs present several advantages in this sense, their potential use might be limited by their significant *in vivo* toxicity and instability, and high costs of production [41–43]. In order to overcome some of these drawbacks, we *de novo* designed, by a minimalistic approach, a series of synthetic amidated lipopeptides with a very short Arg-rich peptide moiety and linked at N-terminus to fatty acids with different lengths, with the aim to find the optimal combination through the variation of hydrophobicity and amino acid composition of the molecule.

The screening software indicated the R**X**WR sequence as optimal template for USCLs. In particular, these amino acid residues are invariably present in many AMP suggesting that two types of side chains are essential for antimicrobial activity. The cationic side chains of Arg, Lys and, less frequently, His mediate peptide interactions with negatively charged bacterial membranes and/or cell walls components, including LPS [44]. Bulky nonpolar side chains, such as Pro, Phe, and Trp, which occur frequently in AMPs, provide lipophilic anchors that ultimately induce membrane disruption [45]. The side chains of Arg and Trp are present in many AMPs with different sizes and secondary structures. For example, Ac-RRWWXX-NH_2_ hexamers derived from synthetic combinatorial libraries are active AMPs [46], while other Arg- and Trp-containing hexapeptides were effective antibacterial agents regardless of the position of both amino acids in the sequence [47].

The bactericidal potency of RFWR tetrapeptide was improved by the addition of an appropriate hydrophobic tail, a strategy that has been often used for the optimization of many antimicrobials [17, 18, 48]. The results of MIC assays with USCLs linked to fatty acids of different length indicated that a C12 chain is the optimal choice to obtain molecules with potent activity towards both Gram-positive *S*. *aureus* and Gram-negative *E*. *coli* and *P*. *aeruginosa*. These results are in agreement with previous studies in which it has been demonstrated that *i*) short lipopeptides with 10 and 12 carbon atoms are generally active towards both bacteria and fungi and are also non-hemolytic compounds [49], and *ii*) that 12–14 carbon atoms constitute the optimal fatty acid length for the best membrane disruption effect [38]. In addition to these, our findings demonstrate that the substitution of the second residue in the R**X**WR peptide template did not drastically modify the antibacterial activity, indicating that **X** position is not critical for the antimicrobial activity of USCLs. These results validated the prediction of the consensus peptide sequence used for the rational design of USCLs.

Many studies describe the antimicrobial activity of lipopeptides containing trimer or tetramer rich in Lys residues [18, 38, 50–52]. The good antimicrobial activity of our Arg-rich USCLs demonstrated that Arg residues in the peptide sequence of lipopeptides are a valid alternative to Lys-rich ones. Moreover, the most active Lp-I showed a better MIC values than those reported for some Lys-rich lipopeptides of equal length of peptide and/or acyl chain [18, 38, 51, 52], thus suggesting a key role of Arg residues for the antimicrobial activity. Arg residues are considered responsible for the preferential interactions of the peptides with negatively charged bacterial membranes. In fact, in some Trp-rich AMPs containing exclusively either Arg or Lys as the positively charged residues, the Arg-to-Lys mutations induced a decrease of antimicrobial activity that was correlated with a decrease in their ability to permeabilize bacterial membranes [53, 54]. Molecular dynamics simulations indicated that Arg can form extensive H-bonding with the phospholipid head groups, enhancing the membrane-disruptive activity [55].

Surface plasmon resonance analysis confirmed the binding capability of Lp-I and Lp-I^RR^ to liposomes mimicking a model of Gram-positive membrane, already predicted by our molecular dynamics study. This result confirms that the prediction obtained by molecular dynamics can be definitely very informative for the *de novo* design of new antibacterial molecules.

As reported for other lipopeptides [6, 18, 19], Lp-I and Lp-I^RR^ act by increasing the permeability of the bacterial membranes. The membranolytic activity of both USCLs, at concentrations near the MIC values, caused a decrease of *S*. *aureus* viability, indicating that membrane lesions were likely lethal for bacterial cells, as also observed by SEM images. Moreover, Lp-I showed a very rapid membrane permeabilization, even at sub MIC concentration, whilst Lp-I^RR^ caused only a partial permeabilization at MIC concentration, indicating that its effect on bacterial viability is likely due not only to a membrane disruption effect, but also to an additional mechanism such as membrane depolarization, as already reported for many cationic lipopeptides [56–59].

Commonly, membrane-active agents represent an important weapon for fighting persistent infections that generally involve slow-growing or non-growing bacteria [60], and in many cases their mechanism of action reduces the rate of resistance development. The effect of these USCLs on bacterial membranes suggest that they could at least contribute to minimize the development of drug resistance. For example, the frequency of phenotypes showing resistance to polymyxins, a family of cyclic membrane-active lipopeptides, is generally less than 10% [61]. In *S*. *aureus*, the development of resistance to the lipopeptide daptomycin, used to treat complicated skin infections [62] remains rare [63]. Unfortunately, these native lipopeptides are non-cell-selective and therefore quite toxic to mammalian cells [11, 12]. On the contrary, here we demonstrated that the Lp-I and Lp-I^RR^ USCLs reduced the cell viability only at concentrations well above the MIC values against bacterial and fungal strains. This results confirmed that, unlike most of the native lipopeptides, these synthetic lipopeptides showed an improved cell selective toxicity, a result in agreement with other studies carried out with different ultrashort lipopeptides [17–19]. However, we observed that Lp-I and Lp-I^RR^ decreased the cell viability to different extents; on this basis we believe that the higher hydrophobicity and the lower positive charge of Lp-I could facilitate its diffusion through the membranes and the epidermis layers, where it affects cell viability at lower concentrations than Lp-I^RR^.

Although the detailed mechanism of action of all the lipopeptides is not completely understood, some key features has emerged [8, 64, 65]. A number of lipopeptides tend to form oligomers, micelles or aggregates [8], depending on their concentrations and conditions in solution (*e*.*g*. presence of Ca^2+^) [65]. In this regard, a membrane-binding model of a synthetic antimicrobial short lipopeptide was proposed by Horn *et* al. [64], providing some insights into a possible mechanism of antimicrobial action by means of a molecular dynamics study. According to this model, C16-KGGK cationic lipopeptide rapidly forms aggregates in solution, thus protecting the acyl chains from water, before affecting the organization of the membrane lipids [64]. Our results obtained with DLS indicate that Lp-I and Lp-I^RR^ are indeed able to produce heterogeneous and polydisperse populations of aggregates in the concentration range of their MIC values (as also suggested by surface plasmon resonance) and at higher concentrations, indicating that different particle sizes of aggregates does not affect the antimicrobial activity neither the cytotoxicity. Moreover, the presence of two additional Arg in Lp-I^RR^ seems to significantly reduce the size of aggregates as compared to Lp-I. The larger size of Lp-I aggregates likely increases the lipopeptide concentration at the cell membrane making this compound more cytotoxic than Lp-I^RR^.

According to the abovementioned mechanism [64], we could speculate that, when the cationic peptide moiety of the lipopeptide aggregate interacts with the anionic phospholipids, the cell surface-bound aggregate does not fully protect the lipophilic tail, causing dissociation and insertion of lipopeptides into the phospholipid bilayer. Although the organization of the lipopeptides, either in solution or bound to a specific cell, is crucial for the interpretation of their biological function, we investigated their aggregation state and biological activities using different experimental settings. Nevertheless, in surface plasmon resonance analysis, the binding curves obtained at 6 and 12 μg/mL evidenced the presence of membrane-bound aggregates for both USCLs. In addition, only Lp-I aggregate is able to disaggregate, whilst Lp-I^RR^ seems to form a more stable aggregate bound to the membrane. This interpretation correlates with the different permeabilizing and growth-inhibiting rate of Lp-I and Lp-I^RR^ that might be ascribed to a different stability of their membrane-bound aggregates and a different kinetics of lipopeptides dissociation and insertion into the bacterial membrane.

This fact has already been demonstrated for palmitoylated antimicrobial lipopeptides, where the substitution of some amino acid residues modified the stability of oligomers, making their dissociation more difficult and thus producing less active molecules [13], and for short acyl-lysine oligomers which displayed dissimilar antibacterial properties due to a different binding to phospholipid membranes, despite their similar tendency to self-assemble in solution [66].

Furthermore, as described for the lipopeptide mimetic oligo-acyl-lysine [66, 67], the highly hydrophilic and anionic external membrane of Gram-negative bacteria is likely to retain cationic aggregates more effectively, thus preventing the lipopeptide progression toward the inner membrane; this fact could explain why both our USCLs displayed overall weaker activities against Gram-negative bacteria as compared to Gram-positive ones.

In conclusion, our approach, originating from rationally designed USCLs, enabled us to create new compounds, characterized by very short Arg-rich peptide sequence, potent antimicrobial activity and high cell selectivity. Considering their mechanism of action, these USCLs could be useful to develop new drugs also effective against slow- or non-growing bacteria, with a limited risk to develop drug resistance, thus making them very promising antimicrobial and/or antifungal agents [18, 19].

## Supporting information

S1 Fig Hemolytic activity of Lp-I and Lp-I^RR^ on human erythrocytes (hRBC). Hemolysis of hRBC was evaluated after 60 min incubation at 37°C with indicated concentrations of Lp-I (light blue bars) and Lp-I^RR^ (green bars). Each value is expressed as the mean ± SEM of three independent experiments carried out in duplicate. *p < 0.0001 *vs* untreated hRBC (Student-Newman-Keuls Multiple Comparisons Test, ANOVA).
(PDF)

S2 Fig Analysis of the aggregation potential of Lp-I and Lp-I^RR^. (**a**) Representative dynamic light-scattering distribution curves of Lp-I^RR^ (red line) and Lp-I (green line) nanoparticles’ hydrodynamic diameters, both measured at the concentration of 100 μM in PBS. (**b**) Mean hydrodynamic diameters of Lp-I^RR^ (red bars) and Lp-I (green bars) aggregates calculated at 10, 50, 100 and 150 μM. Each bar value derived from three acquisitions; dimensional data originating from minor aggregate populations were omitted. Intensity-weighted size distributions (see panel **a**) were used to extract the mean diameter of the nanoparticles reported in the bar chart at different concentrations. *p < 0.05 *vs* size of Lp-I aggregates at the same concentration (Student-Newman-Keuls Multiple Comparisons Test, ANOVA).
(PDF)

S3 Fig Molecular dynamics. Snapshot of molecular dynamics at T0, T25 ns and T50 ns of Lp-I (**a**) and Lp-I^RR^ (**b**); yellow spheres represent sodium (Na^+^), the red ones represent chlorine (Cl^-^). The enlarged image in panel (**c**) shows the interaction of Lp-I with membrane model of *S*. *aureus* at 200 ns. Red dashed-lines represent the hydrogen bonds. The heterogeneous *S*. *aureus* bilayers was modeled following the CHARMM-GUI Membrane Builder step-by-step protocol [J Comput Chem (2008) 29: 1859–1865; Biophys J (2009) 97: 50–58]. The force-field parameters for each lipid were assigned from the CHARMM36 force field [J Phys Chem B (2010) 114: 7830–7843]. The system contains 240 POPG molecules (120 in each leaflet), 120 of TOCL2 (Cardiolipin) (120 in each leaflet), in 150 mM NaCl. The number of atoms in the system are ~112000. Three replicas for each bilayer system were built to improve sampling and to check simulation convergence.
(PDF)

S4 Fig Evaluation of membrane-damaging activity of Lp-I and Lp-I^RR^ on *E. coli* ATCC 25922 by PI-uptake assay. The permeabilization assay with Lp-I (**a**) and Lp-I^RR^ (**b**) on *E*. *coli* ATCC 25922 cells has been performed in MHB. Bacterial cells (1×10^6^ CFU/mL) were incubated, for different incubation times, with USCLs at the concentration equal to their MIC, ½MIC or 2×MIC. % PI-positive: percentage of propidium iodide positive cells. The background level of permeabilized cells, obtained using untreated samples, was always lower than 2% and was subtracted to the corresponding USCL-treated sample. Data are a mean ± SEM of four independent experiments.
(PDF)

S5 Fig USCLs effect on *S. aureus* ATCC 25923 viability. The bactericidal activity of Lp-I (light blue bars) and Lp-I^RR^ (green bars) on *S*. *aureus* was determined using a mid-logarithmic phase bacterial suspension, diluted in fresh MHB to a final concentration of 1×10^6^ CFU/mL, and incubated at 37°C with indicated concentrations of USCLs. After 30 min incubation, samples were removed, diluted in PBS, plated on MH agar and incubated overnight to allow the colony counts. Data are a mean ± SEM of three independent experiments. *p < 0.05 *vs* untreated cells (ctrl, white bars), **p ≤ 0.005 *vs* untreated cells (ctrl, white bars) (ANOVA with post-test Tukey-Kramer).
(PDF)

## Abbreviations

USCLs: ultra-short cationic lipopeptides
MRSA: methicillin-resistant *S*. *aureus*
AMP: antimicrobial peptides
PI: Propidium Iodide
DLS: Dynamic Light Scattering
LUVs: Large unilamellar vesicles
DPPC: dipalmitoylphosphatidylcholine
DPPG: 1,2-dipalmitoylphosphatidylglycerol
RU: Relative Units
VREF: *Enterococcus faecalis* vancomycin-resistant
GEN: gentamicin
IPM: imipenem
OXA: oxacillin
PEN: penicillin
CIP: ciprofloxacin
CEF: cephalothin
CLI: clindamycin
ERY: erythromycin
AMK: amikacin
ATM: aztreonam
NET: netilmicin
TEC: teicoplanin
TET: tetracycline
TOB: tobramycin
VAN: vancomycin
MHB: Mueller-Hinton Broth
SAB: Sabouraud dextrose
CLSI: Clinical & Laboratory Standards Institute
CFU: colony-forming unit
DMEM: Dulbecco’s Modified Eagle’s Medium
FBS: fetal bovine serum
