# Supplementary figures and images for: Design, antimicrobial activity and mechanism of action of Arg-rich ultra-short cationic lipopeptides
Source: PLoS One. 2019 Feb 21;14(2):e0212447. doi: 10.1371/journal.pone.0212447 (PMC6383929; doi:10.1371/journal.pone.0212447)

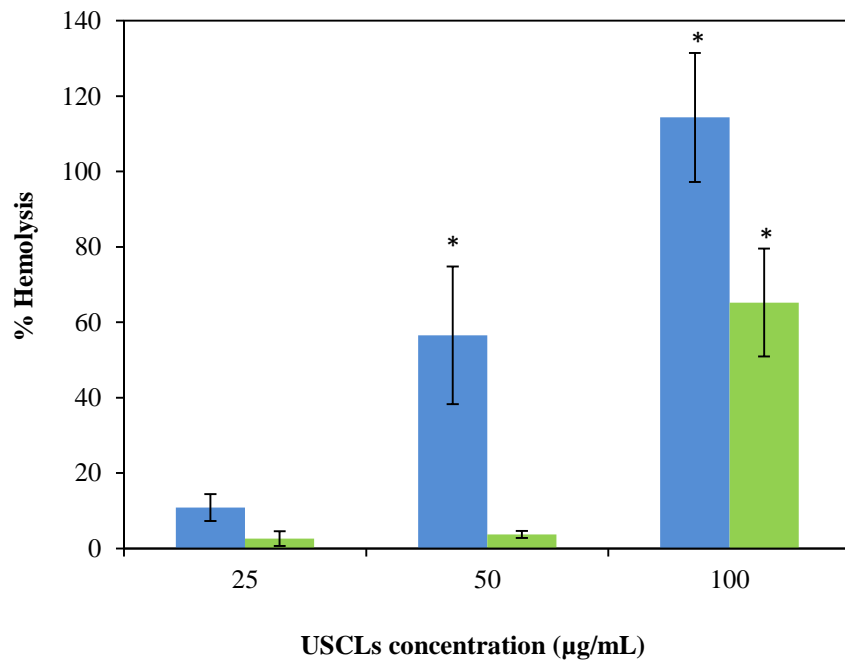

Supplement: S1 Fig — Hemolysis of hRBC was evaluated after 60 min incubation at 37°C with indicated concentrations of Lp-I (light blue bars) and Lp-IRR (green bars). Each value is expressed as the mean ± SEM of three independent experiments carried out in duplicate. *p < 0.0001 vs untreated hRBC (Student-Newman-Keuls Multiple Comparisons Test, ANOVA). (PDF) [file pone.0212447.s001.pdf]

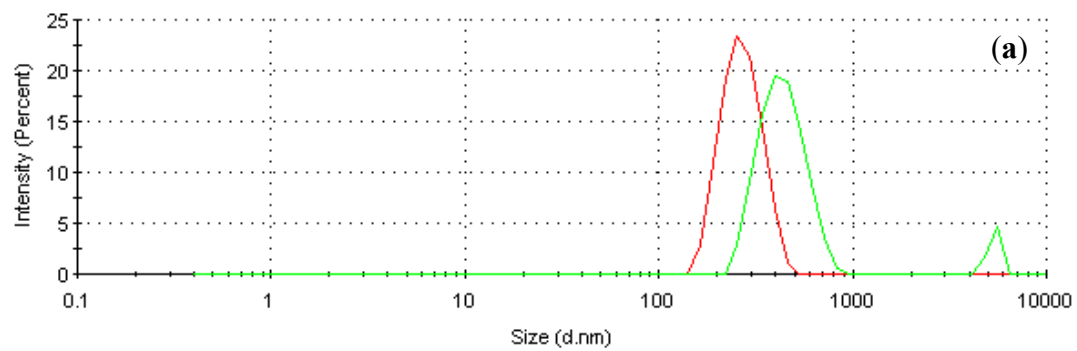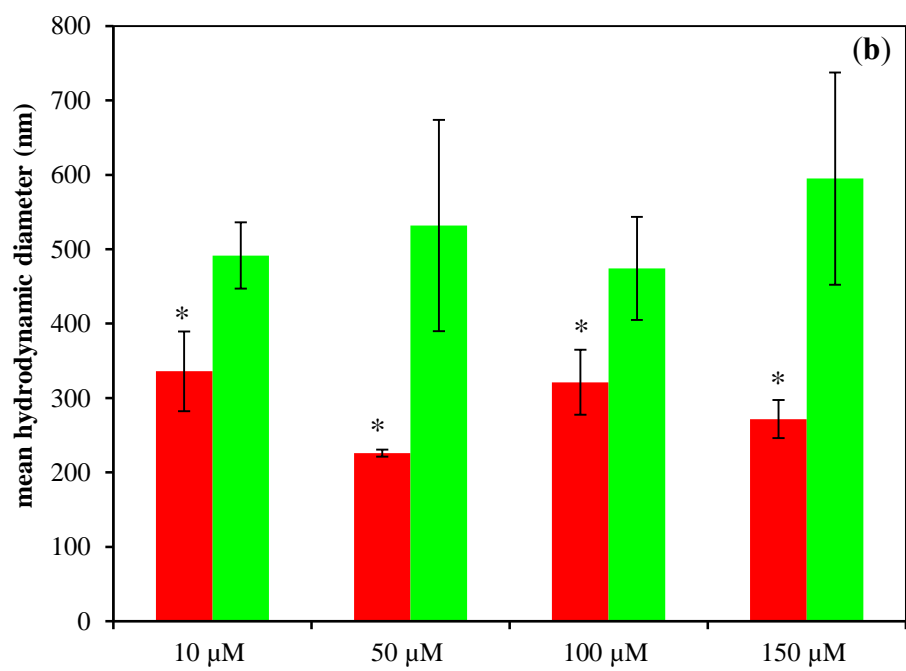

Supplement: S2 Fig — (a) Representative dynamic light-scattering distribution curves of Lp-IRR (red line) and Lp-I (green line) nanoparticles’ hydrodynamic diameters, both measured at the concentration of 100 μM in PBS. (b) Mean hydrodynamic diameters of Lp-IRR (red bars) and Lp-I (green bars) aggregates calculated at 10, 50, 100 and 150 μM. Each bar value derived from three acquisitions; dimensional data originating from minor aggregate populations were omitted. Intensity-weighted size distributions (see panel a) were used to extract the mean diameter of the nanoparticles reported in the bar chart at different concentrations. *p < 0.05 vs size of Lp-I aggregates at the same concentration (Student-Newman-Keuls Multiple Comparisons Test, ANOVA). (PDF) [file pone.0212447.s002.pdf]

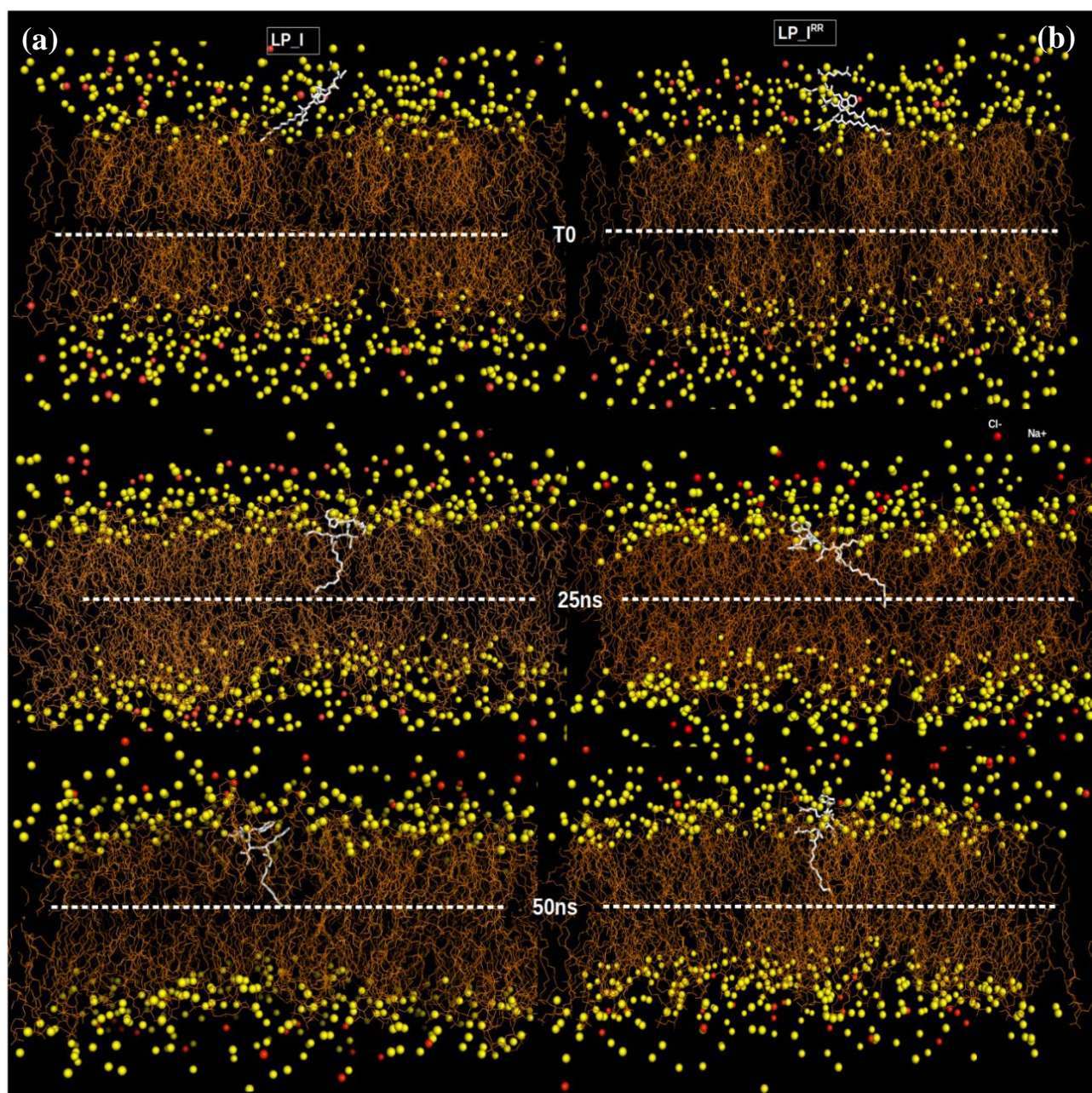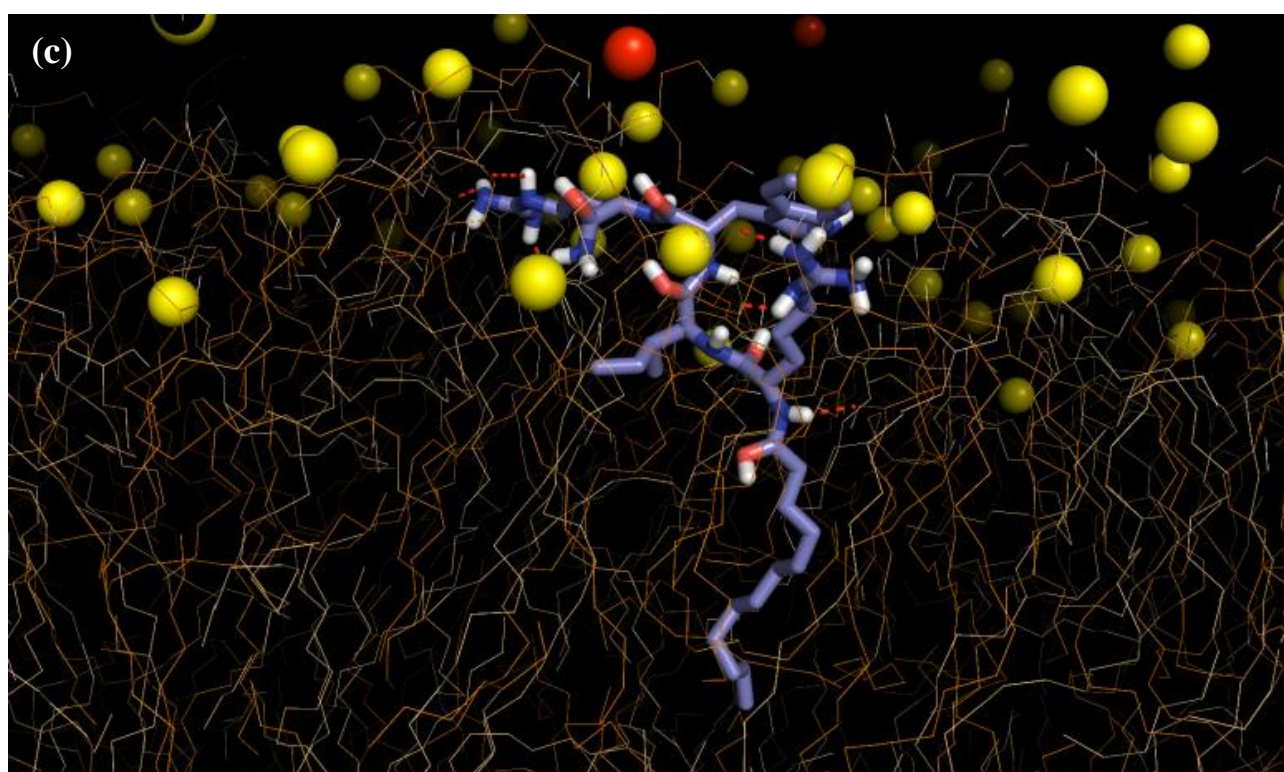

Supplement: S3 Fig — Snapshot of molecular dynamics at T0, T25 ns and T50 ns of Lp-I (a) and Lp-IRR (b); yellow spheres represent sodium (Na+), the red ones represent chlorine (Cl-). The enlarged image in panel (c) shows the interaction of Lp-I with membrane model of S. aureus at 200 ns. Red dashed-lines represent the hydrogen bonds. The heterogeneous S. aureus bilayers was modeled following the CHARMM-GUI Membrane Builder step-by-step protocol [J Comput Chem (2008) 29: 1859–1865; Biophys J (2009) 97: 50–58]. The force-field parameters for each lipid were assigned from the CHARMM36 force field [J Phys Chem B (2010) 114: 7830–7843]. The system contains 240 POPG molecules (120 in each leaflet), 120 of TOCL2 (Cardiolipin) (120 in each leaflet), in 150 mM NaCl. The number of atoms in the system are ~112000. Three replicas for each bilayer system were built to improve sampling and to check simulation convergence. (PDF) [file pone.0212447.s003.pdf]

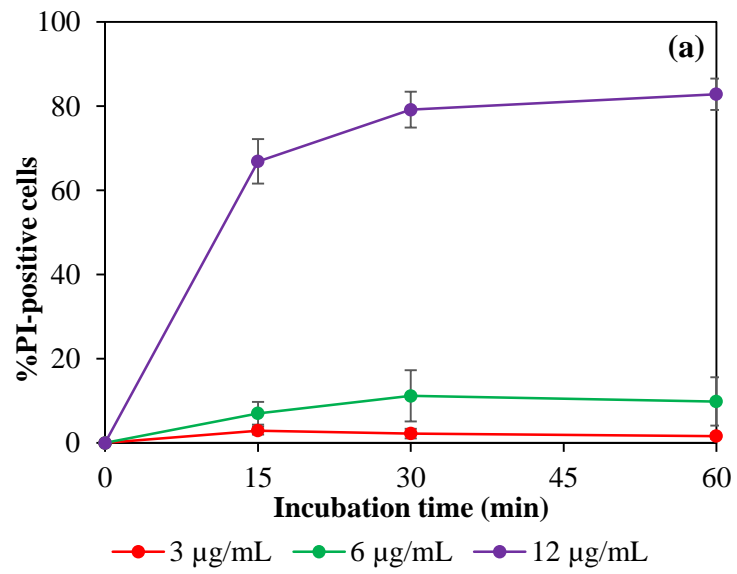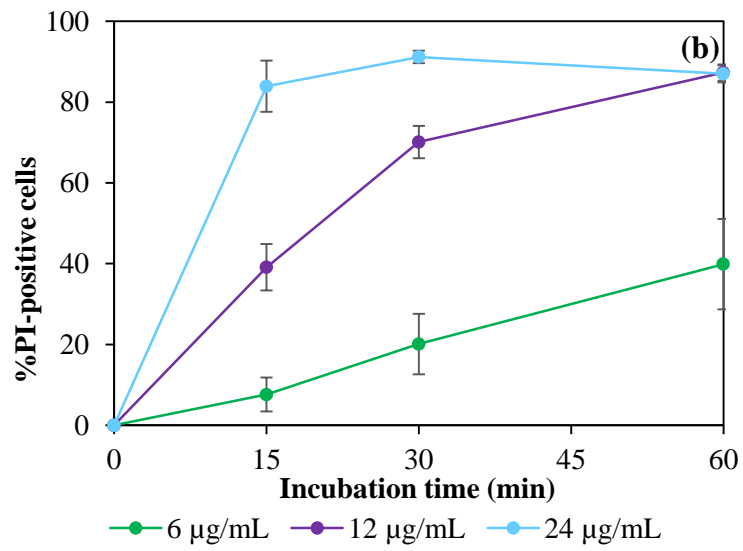

Supplement: S4 Fig — The permeabilization assay with Lp-I (a) and Lp-IRR (b) on E. coli ATCC 25922 cells has been performed in MHB. Bacterial cells (1×10^6^ CFU/mL) were incubated, for different incubation times, with USCLs at the concentration equal to their MIC, ½MIC or 2×MIC. % PI-positive: percentage of propidium iodide positive cells. The background level of permeabilized cells, obtained using untreated samples, was always lower than 2% and was subtracted to the corresponding USCL-treated sample. Data are a mean ± SEM of four independent experiments. (PDF) [file pone.0212447.s004.pdf]

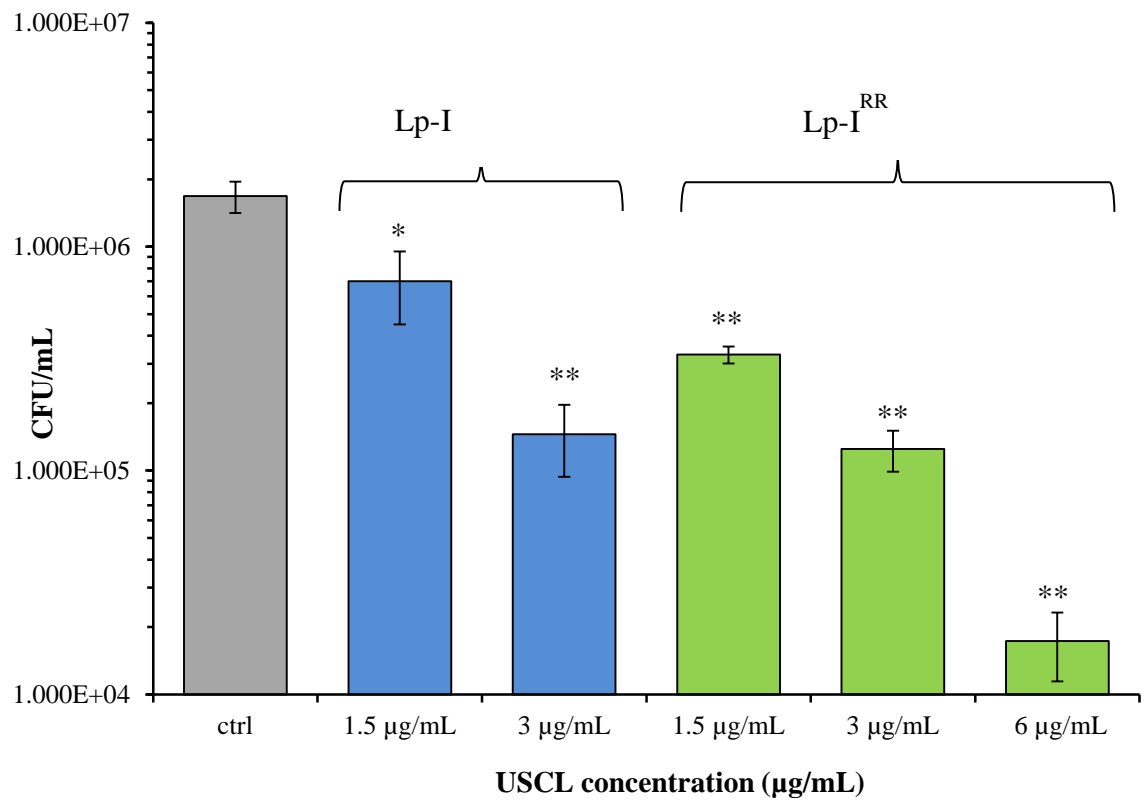

Supplement: S5 Fig — The bactericidal activity of Lp-I (light blue bars) and Lp-IRR (green bars) on S. aureus was determined using a mid-logarithmic phase bacterial suspension, diluted in fresh MHB to a final concentration of 1×10^6^ CFU/mL, and incubated at 37°C with indicated concentrations of USCLs. After 30 min incubation, samples were removed, diluted in PBS, plated on MH agar and incubated overnight to allow the colony counts. Data are a mean ± SEM of three independent experiments. *p < 0.05 vs untreated cells (ctrl, white bars), **p ≤ 0.005 vs untreated cells (ctrl, white bars) (ANOVA with post-test Tukey-Kramer). (PDF) [file pone.0212447.s005.pdf]
